# Supplementary material for: Chromothripsis during telomere crisis is independent of NHEJ, and consistent with a replicative origin
Source: Genome Res. 2019 May;29(5):737–49. doi: 10.1101/gr.240705.118 (PMC6499312; doi:10.1101/gr.240705.118)
Supplement: Supplemental Material [file supp_gr.240705.118_Supplemental_file_1.zip › contigs/annotated_contigs/DB82/contig.3.DB82_length_360_mean_cov_10.9416666667.docx]

**DB82_length_360_mean_cov_10.9416666667**

```
CAATGATATTAAGGTCTAGTTGACTTCACATCCTCTATTCGTTTATTTTCCTTCTTTGTCTTACTTCCCCACTATGCTAATAGTATTTT
 >chr10:64777091-64777241 - E=7e-79
TTGGAATAACTTTCCAAATAAAACTATTTGCACCTGAATTATTTTCTGAAGGTCGGCAT|CT|AAAAACACTTCTCAAGGTTTGGCAAT
                                                            >chr10:64775085-64775155 -
CACTTCATCACTTCTATGGAGATCATCTAGTCCAGTTATCTT|GA|CTGGGCACGGTGGCTCACGCCTGTAATCCCAGCACTTTGGGAG
E=1e-30                                      >chr15:27004563-27004703 - E=8e-64
GCCGAGGCGGGTGGATCACGAGGTCAGGAGTTCGAGACCAGCCTGGCCAACATGGTGAAACCCCGTCTCTACTAAAAATACAAAAATTA

GCTGGGCA
```
